# Supplementary material for: Passive performance evaluation and validation of a viscous impeller pump for subpulmonary fontan circulatory support
Source: Sci Rep. 2023 Aug 4;13:12668. doi: 10.1038/s41598-023-38559-y (PMC10403595; doi:10.1038/s41598-023-38559-y)
Supplement: Supplementary file 1 — Supplementary Information. [file 41598_2023_38559_MOESM1_ESM.pdf]

Supplementary Materials

Mesh Sensitivity Study

To assess the impact of mesh size on pressure loss, a mesh sensitivity study was performed on the baseline VIP design with four mesh refinement levels (coarse, intermediate, fine and very fine). The cardiac output tested on the mesh sensitivity study was set to 11 L/min. For the coarse mesh, the general isotropic mesh size and the tubing surface mesh size were set to 0.4 and 0.12 cm respectively. The surface mesh size for impellers and struts was reduced to 0.04 cm and 0.08 respectively. We attached boundary layer elements to the wall where the mesh size at the n-th innermost layer is $\frac{1}{2^n}$ of the general isotropic mesh size. To reduce computational cost, n was set to 3 and 1 for the central test section and peripheral tubing, respectively. The resulting coarse mesh consists of 3.8 million linear tetrahedra and 705k nodes. For the intermediate mesh, the general isotropic mesh size was reduced to 0.2 cm while the surface mesh parameters for the test section were reduced by 30%, resulting in 7.7 million elements and 1.4 million nodes. For the fine mesh, the general mesh size and surface mesh size were further reduced by 50% and 30%, respectively, resulting in 14 million elements and 2.7 million nodes. Then, a local mesh refinement was performed to reduce the max mesh size to 0.02 cm for the test section (up to 5 cm away from the origin) resulting in a very fine mesh of 31 million elements and 5.7 million nodes.

Figure S1 shows the mesh density and element aspect ratio near the pump equator at fours refinement levels. Figures S2 compares the velocity field in the test section. Compared to the pressure loss ($\Delta P = 2.34$ mmHg) obtained from the very fine mesh, ΔP was 2.34, 2.30 and 2.36 mmHg with the coarse, intermediate and fine meshes, respectively. The differences in pressure loss were within 3%. Therefore, the mesh parameters for the intermediate mesh were used for the other models and flow conditions due to a reduced surface mesh size and an improved velocity field without increasing computational cost significantly.

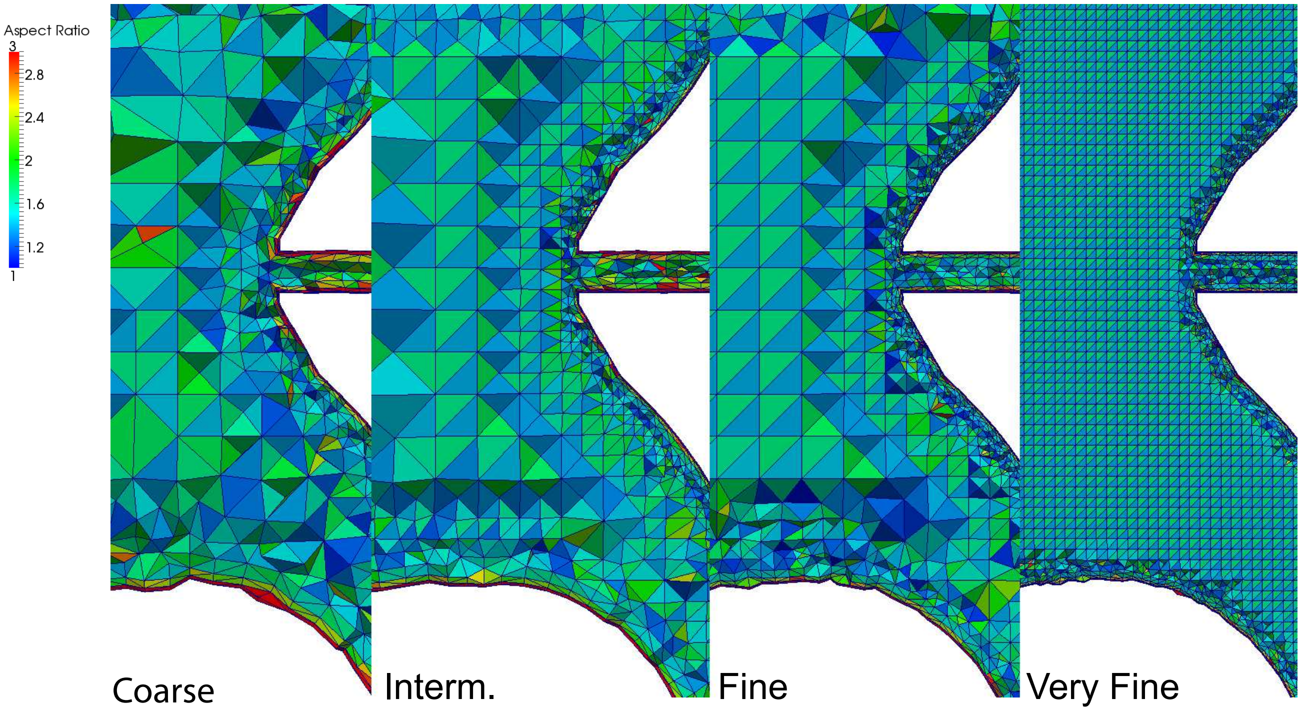

Figure S1. Comparison of four mesh refinement levels for a baseline VIP model. A plane clip cut is created to show the element aspect ratio and mesh density.

Model	Aspect Ratio	Min Angle	Shape
Blank	1.97 (0.79)	40.15 (17.11)	0.70 (0.16)
1.09 mm	2.14 (5.44)	39.55 (17.79)	0.69 (0.17)
1.62 mm	2.07 (1.02)	39.43 (17.85)	0.69 (0.17)

Table S1. Mesh quality metrics are shown as mean (standard deviation). The quality metrics were computed by the Verdict Geometric Quality Library[1].

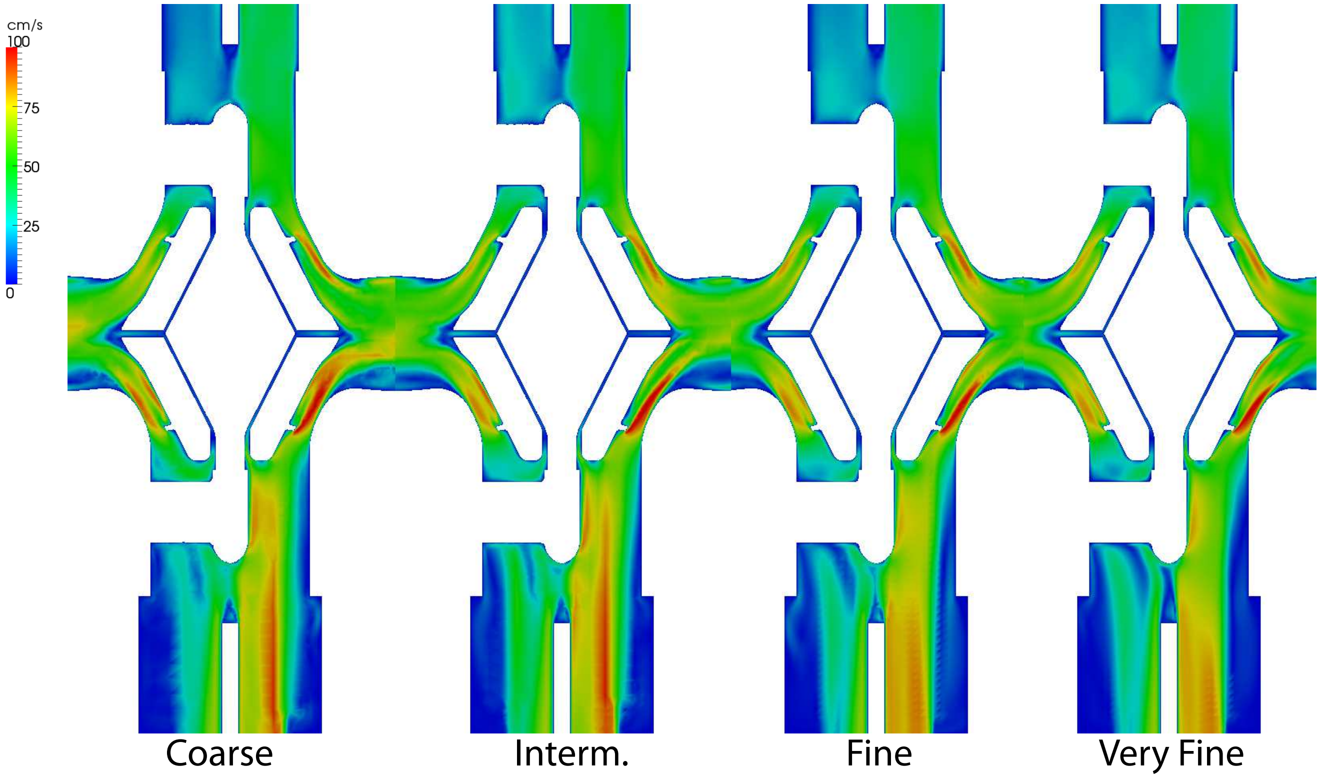

Figure S2. Mean velocity magnitude fields on a central cut plane with four mesh refinement levels.

Mesh quality metrics (aspect ratio, minimum dihedral angle and shape) for the intermediate mesh level are summarized in Table S1.

Additional Figures

Power loss was also calculated based on the control volume bounded by the 4 cut planes at 4 cm away from the origin,

$$E_{diss} = \sum_{i=1}^{2} Q_i \left(P_i + \frac{1}{2}\rho V_i^2 \right) - \sum_{i=3}^{4} Q_i \left(P_i + \frac{1}{2}\rho V_i^2 \right), \tag{1}$$

where Q_i, P_i and V_i are averaged flow rate, pressure and velocity, respectively, on cut plane i, which is 4 cm away from the origin in the upstream (i=1 and 2) or downstream (i=3 and 4).

Figure S3 demonstrates that VIPs at 0 RPM do not create significant energy loss compared to the Fontan procedure.

Figure S4 shows TAWSS on the housing surface. The blank housing model resulted in a lower WSS level and an increased region of low WSS on the housing surface compared to the models with a VIP inside (Figure S4). OSI on the rotor and strut surfaces was nearly zero for CO $\leq$ 5 L/min. The size of the region with non-zero OSI increased with increasing CO due to flow separation past the rotor blades and struts (Figure S5). In contrast, the flow in the secondary path was nearly unidirectional regardless of cardiac output.

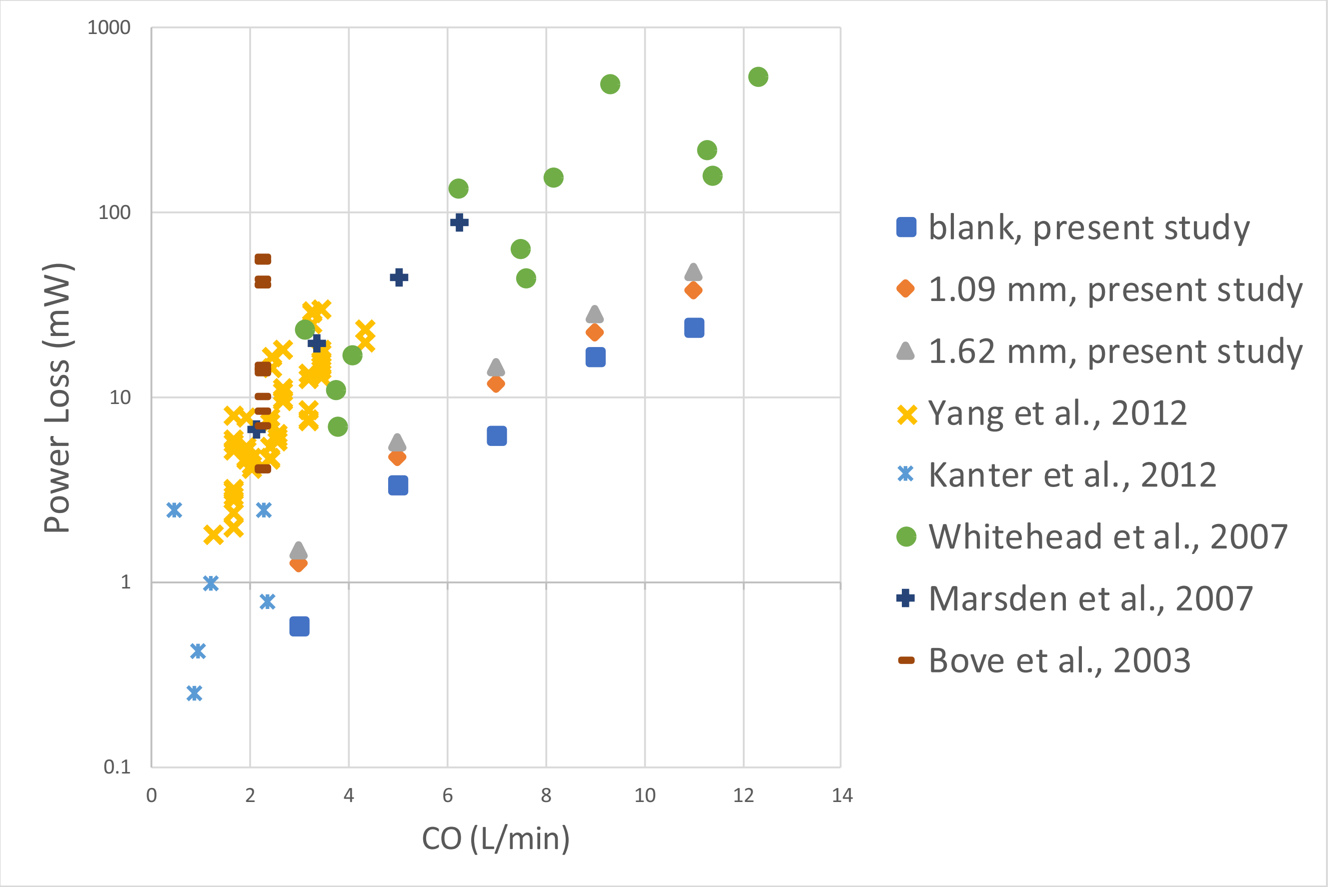

Figure S3. Power loss for a blank pump housing, a static pump with two different blade heights, and representative Fontan models reported by Yang et al.[2], Kanter et al.[3], Whitehead et al.[4], Marsden et al.[5], and Bove et al.[6]. Note that the ordinate is in a base-10 log scale.

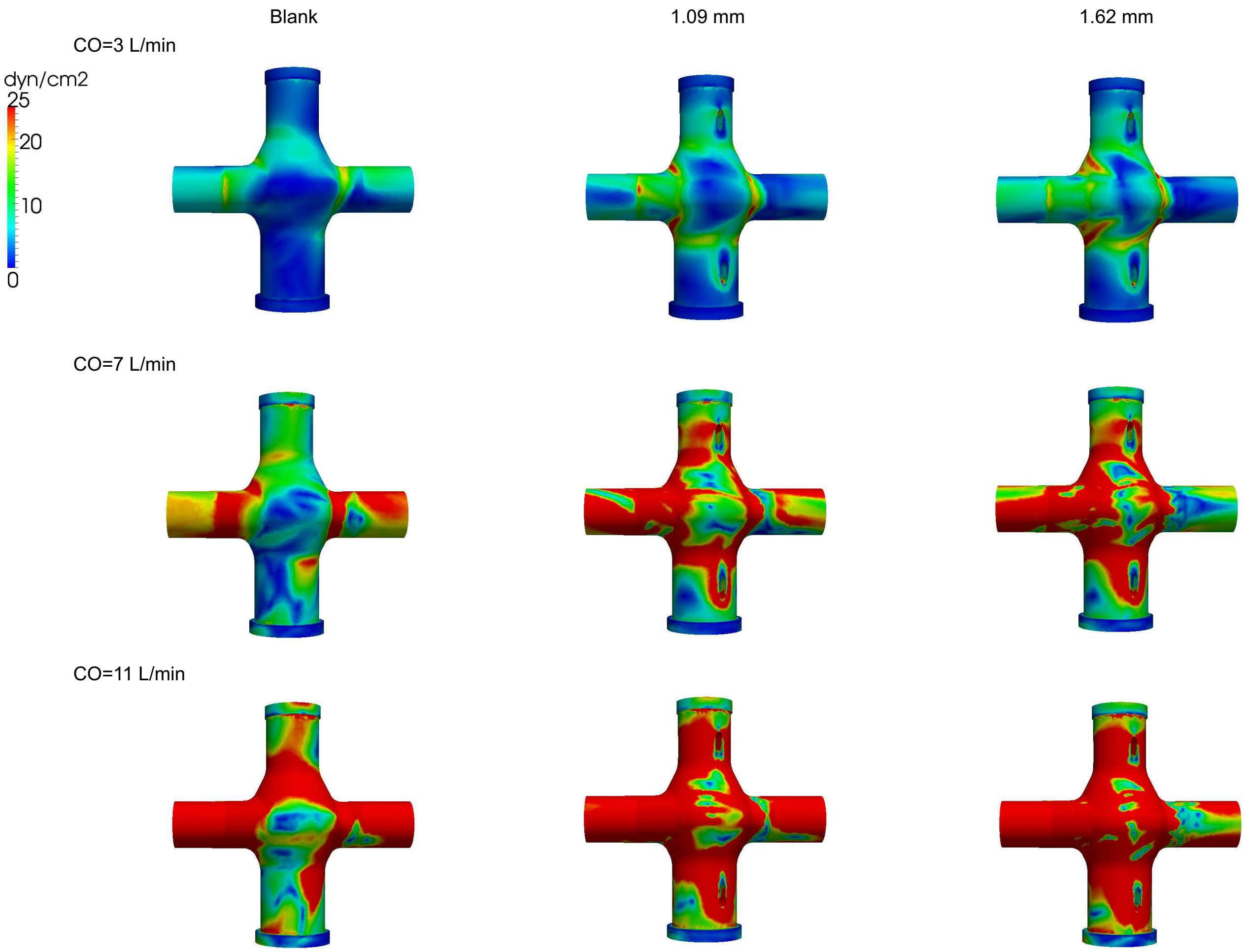

Figure S4. Time averaged wall shear stress (TAWSS) distribution on the housing surface with and without a pump inside.

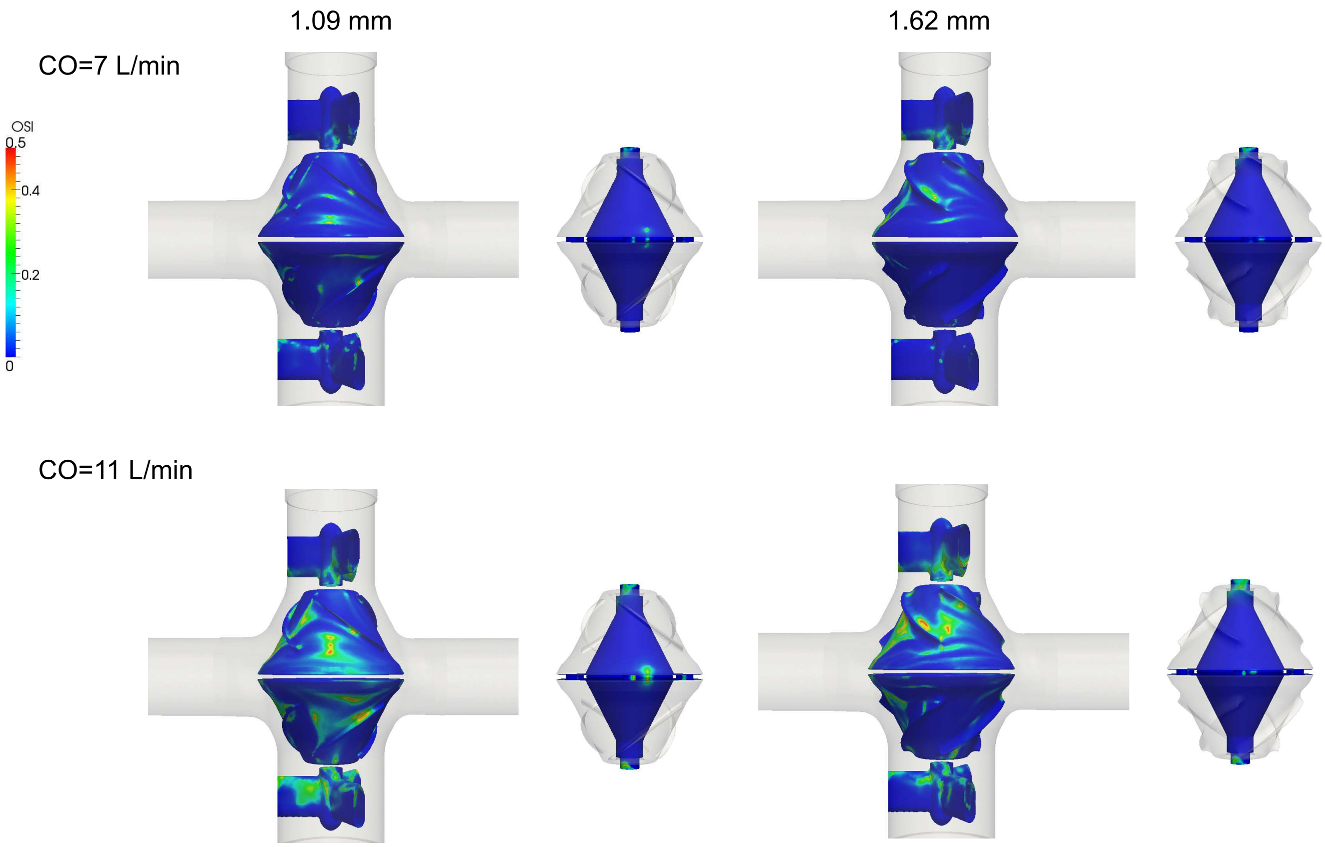

Figure S5. Oscillatory shear index (OSI) on the rotor and secondary flow surfaces at CO=7 and 11 L/min. OSI values on the rotor and strut surfaces increase with increasing CO values due to flow separation while the flow in the secondary flow was unidirectional leading to low OSI.

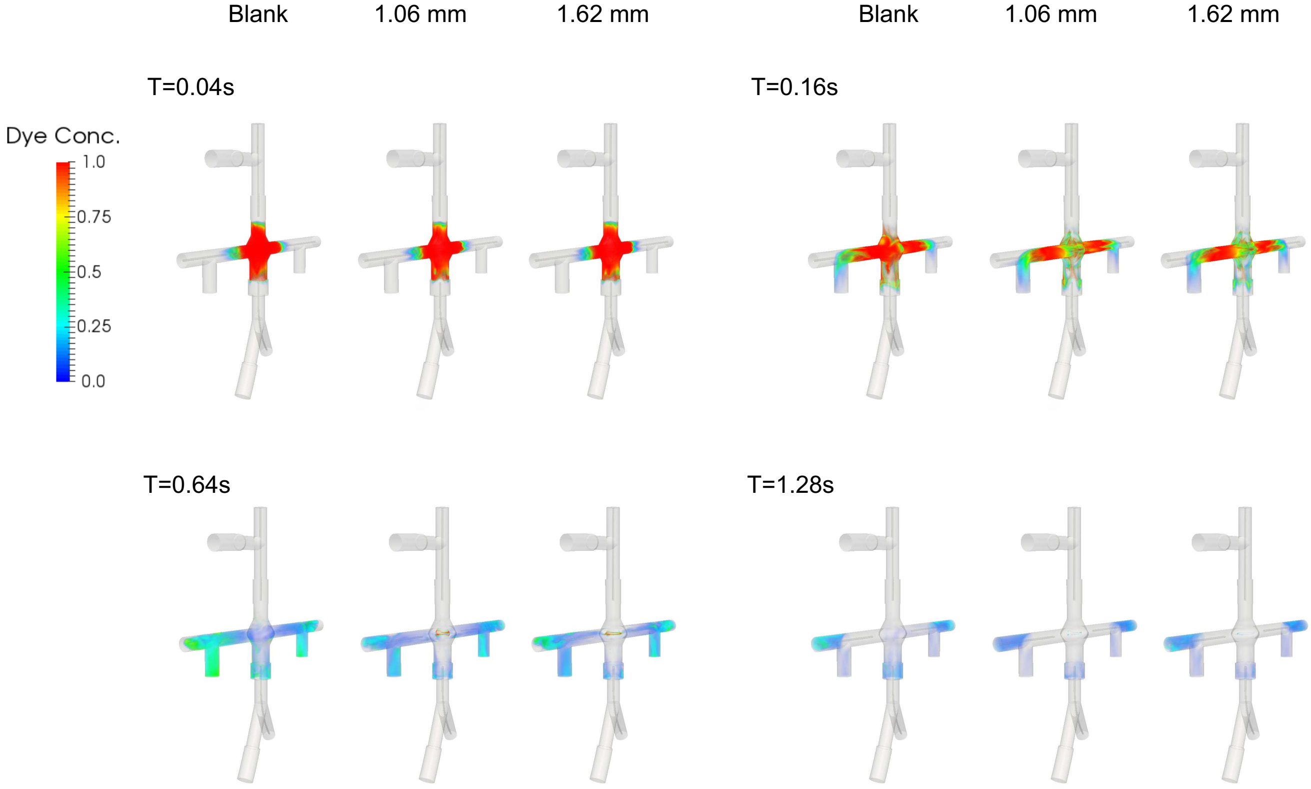

Figure S6. Dye concentration at T =0.04, 0.16, 0.64s for a blank housing, 1.09 mm blades and 1.62 mm blades at CO=7 L/min. Virtual dyes with $\phi = 1$ were placed in the center of the pump at $T = 0$ and advected with flow.

Figures S6 shows the dye concentration at T =0.04, 0.16, 0.64s for a $\times$ 3.5 $\times$ 3.5 cm cube of virtual dyes initially released at the center of the pump for CO=7 L/min. Figure S7 shows the dye washout time for dyes on a central cut plane at 3 and 7 L/min.

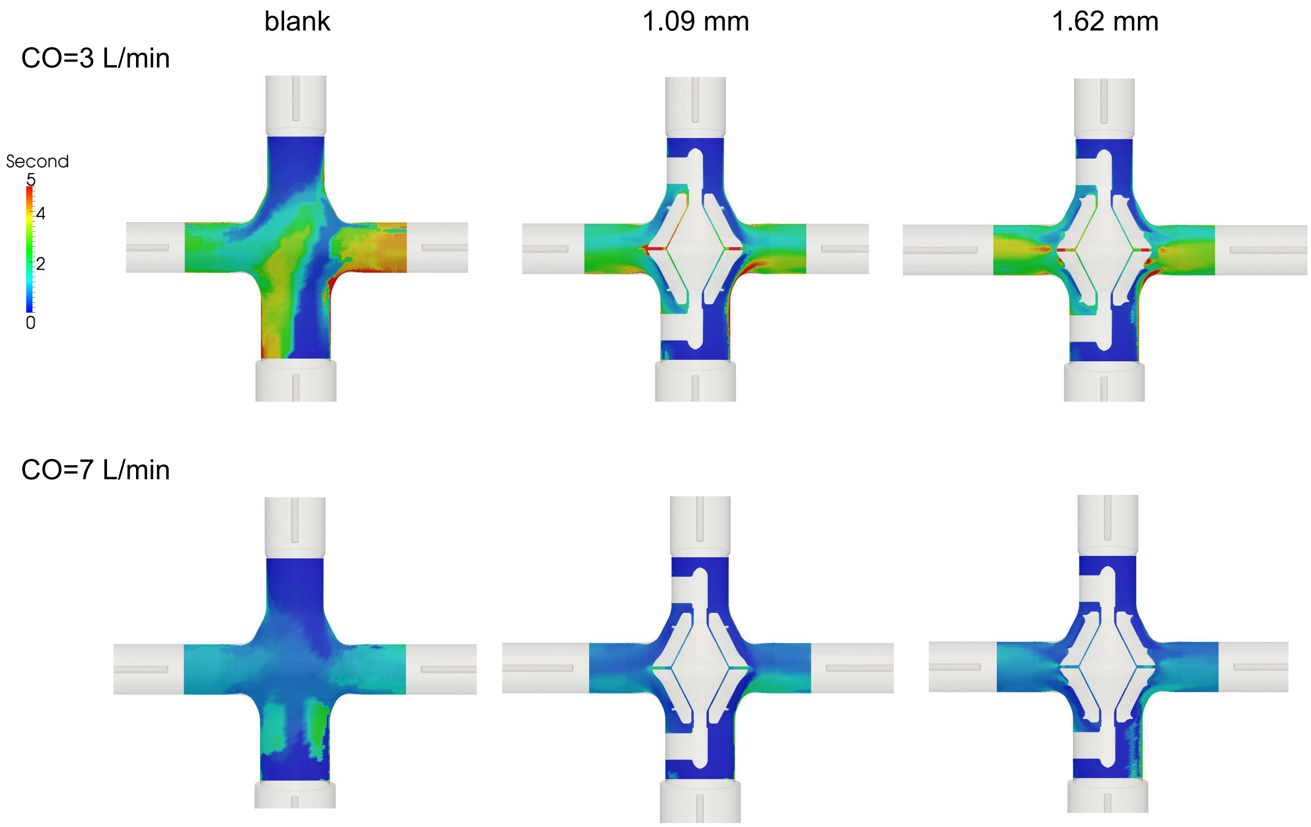

Figure S7. Washout time for dyes on a central cut plane under a cardiac output of 3L/min and 7L/min.

References

1. Knupp, P. M., Ernst, C. D., Thompson, D. C., Stimpson, C. J. & Pebay, P. P. The verdict geometric quality library. Tech. Rep. (2006). DOI: 10.2172/901967.
2. Yang, W. *et al.* Hepatic blood flow distribution and performance in traditional and Y-graft Fontan geometries: A case series computational fluid dynamics study. *J. Thorac. Cardiovasc. Surg.* **143**, 1086–1097 (2012).
3. Kanter, K. R. *et al.* Preliminary clinical experience with a bifurcated Y-graft Fontan procedure- a feasibility study. *J. Thorac. Cardiovasc. Surg.* **144**, 383–389 (2012).
4. Whitehead, K. K. *et al.* Nonlinear power loss during exercise in single-ventricle patients after the Fontan: insights from computational fluid dynamics. *Circulation* **116**, I–165 – I–171 (2007).
5. Marsden, A. L., Vignon-Clementel, I. E., Chan, F., Feinstein, J. A. & Taylor, C. A. Effects of exercise and respiration on hemodynamic efficiency in CFD simulations of the total cavopulmonary connection. *Ann. Biomed. Eng.* **35**, 250–263 (2007).
6. Bove, E. L., de Leval, M. R., Migliavacca, F., Guadagni, G. & Dubini, G. Computational fluid dynamics in the evaluation of hemodynamic performance of cavopulmonary connections after the Norwood procedure for hypoplastic left heart syndrome. *J. Thorac. Cardiovasc. Surg.* **126**, 1040–1047 (2003).
